# Erratum to: KCTU randomisation and IMP management system

**DOI:** 10.1186/s13063-015-0870-3

**Published:** 2015-08-08

**Authors:** Caroline Murphy, Joanna Kelly, John Hodsoll, Evangelos Georgiou, Lloyd Morgan, Christopher Rowson, Victoria Cole, Fatima Jichi, Andrew Pickles

**Affiliations:** King’s Clinical Trials Unit at KHP, Institute of Psychiatry, King’s College London, London, UK; Biomedical Research Centre for Mental Health, South London and Maudsley NHS Trust, London, UK; Biostatistics Group, University College London Hospitals/University College London Research Support Centre, University College London, Gower St., London, WC1E 6BT UK

## Erratum

After publication of this work [1], we noted that we inadvertently failed to include the complete list of all coauthors. The full list of authors has now been added. We are publishing this erratum to update the author list, which is as follows:

Caroline Murphy, Joanna Kelly, John Hodsoll, Evangelos Georgiou, Lloyd Morgan, Christopher Rowson, Victoria Cole, Fatima Jichi, and Andrew Pickles.
